# An Integrative Sialomic Analysis Reveals Molecules From *Triatoma sordida* (Hemiptera: Reduviidae)

**DOI:** 10.3389/fcimb.2021.798924

**Published:** 2022-01-03

**Authors:** Yanna Reis Praça, Paula Beatriz Santiago, Sébastien Charneau, Samuel Coelho Mandacaru, Izabela Marques Dourado Bastos, Kaio Luís da Silva Bentes, Sofia Marcelino Martins Silva, Waldeyr Mendes Cordeiro da Silva, Ionizete Garcia da Silva, Marcelo Valle de Sousa, Célia Maria de Almeida Soares, José Marcos Chaves Ribeiro, Jaime Martins Santana, Carla Nunes de Araújo

**Affiliations:** ^1^ Pathogen-Host Interface Laboratory, Department of Cell Biology, University of Brasilia, Brasilia, Brazil; ^2^ Programa Pós-Graduação em Ciências Médicas, Faculty of Medicine, University of Brasilia, Brasilia, Brazil; ^3^ Laboratory of Protein Chemistry and Biochemistry, Department of Cell Biology, University of Brasilia, Brasilia, Brazil; ^4^ Federal Institute of Goiás, Formosa, Brazil; ^5^ Department of Parasitology, Federal University of Goiás, Goiânia, Brazil; ^6^ Laboratório de Biologia Molecular, Instituto de Ciências Biológicas, Universidade Federal de Goiás, Goiânia, Brazil; ^7^ Laboratory of Malaria and Vector Research, National Institute of Allergy and Infectious Diseases, Bethesda, MD, United States; ^8^ Faculty of Ceilândia, University of Brasilia, Brasilia, Brazil

**Keywords:** triatomine, salivary glands, sialome, sialotranscriptome, sialoproteome, salivary molecules, blood-feeding

## Abstract

Triatomines have evolved salivary glands that produce versatile molecules with various biological functions, including those leading their interactions with vertebrate hosts’ hemostatic and immunological systems. Here, using high-throughput transcriptomics and proteomics, we report the first sialome study on the synanthropic triatomine *Triatoma sordida*. As a result, 57,645,372 reads were assembled into 26,670 coding sequences (CDS). From these, a total of 16,683 were successfully annotated. The sialotranscriptomic profile shows Lipocalin as the most abundant protein family within putative secreted transcripts. Trialysins and Kazal-type protease inhibitors have high transcript levels followed by ubiquitous protein families and enzyme classes. Interestingly, abundant trialysin and Kazal-type members are highlighted in this triatomine sialotranscriptome. Furthermore, we identified 132 proteins in *T. sordida* salivary gland soluble extract through LC-MS/MS spectrometry. Lipocalins, Hemiptera specific families, CRISP/Antigen-5 and Kazal-type protein inhibitors proteins were identified. Our study provides a comprehensive description of the transcript and protein compositions of the salivary glands of *T. sordida*. It significantly enhances the information in the Triatominae sialome databanks reported so far, improving the understanding of the vector’s biology, the hematophagous behaviour, and the Triatominae subfamily’s evolution.

## 1 Introduction

Hematophagous arthropod vectors evolved salivary bioactive molecules that impair and/or modulate prey’s physiological responses triggered by the bite to obtain a blood meal successfully. These proteins have specific and critical targets in the vertebrate host immune and hemostatic systems, acting locally efficiently to obtain blood in a fluid state (Ribeiro, 1995). In recent years, researchers have been investigating the rich mix of the salivary content of arthropod vector species using high-throughput methods, and thus describing the sialomes (mRNA sequences from the salivary gland tissue through transcriptomics + identification of soluble salivary proteins through proteomics) (Santiago et al., 2020a). Vasodilators, antiplatelet, anti-clotting, and anti-inflammatory molecules as well as immunomodulators have been described, comprising a cocktail of molecules that work together in a coordinated and redundant manner for efficacious feeding.

Triatomines or kissing bugs (Hemiptera: Reduviidae) are the insect vectors of the flagellate protozoan *Trypanosoma cruzi*, the etiologic agent of Chagas disease, a Neglected Tropical Disease that causes significant social and economic impacts on Latin America (WHO, 2021). In endemic countries, the main routes of *T. cruzi* transmission occur through the vector (vector-borne) or contaminated food supply (food-borne) when in contact with faeces/urine of an infected triatomine bug. However, alternative routes such as organ transplantation, blood transfusion, and congenital are also possible, which enabled the disease to cross endemic boundaries due to migratory flows in the last decades, making it a worldwide threat (Lindoso and Lindoso, 2009; OPAS, 2017; WHO, 2021). The Triatominae vectors are widely distributed in Latin America, and *Panstrongylus, Rhodnius*, and *Triatoma* genera deserve considerable attention due to their epidemiological significance (Monteiro et al., 2018; Vieira et al., 2018). The *Triatoma sordida* (Stäl, 1859) species presents an extensive geographic distribution, being reported in Argentina, Bolivia, Brazil, Paraguay, and Uruguay (Pacheco de Souza et al., 1978; Forattini, 1980; Panzera et al., 2015; Belintani et al., 2020). The species occupies sylvatic, peridomestic and domestic habitats. In Brazil, *T. sordida* is a synanthropic species commonly captured in human dwellings, revealing its epidemiological importance (Diotaiuti et al., 1998; Noireau et al., 1998; Noireau et al., 1999a; Noireau et al., 1999b; Gurgel-Gonçalves et al., 2012; Galvão and Gurgel-gonçalves, 2014).

Here, we provide a comprehensive description of the sialome of *T. sordida* using RNA-seq and mass spectrometry. The generated data is available at the National Center for Biotechnology Information (NCBI) and Proteome Identifications Database (PRIDE). The database obtained with the assembled coding sequences (CDS) from the transcriptome was used for the proteomic analysis of *T. sordida* salivary gland (SG) soluble extract, with agreement between transcriptome and proteome expression levels. The enrichment of sialome databases reveals that a deep knowledge of the saliva diversity from hematophagous vectors helps to understand vector’s physiology and vector-host interactions as well as the evolutionary mechanisms leading to the insect’s adaptation to the blood-feeding behavior. In addition, the description of these pharmacologically active compounds is of great interest for the development of new approaches for the discovery of anti-thrombotic drugs.

## 2 Materials and Methods

### 2.1 Triatomines and Sample Collection

Field-caught *T. sordida* specimens (adults and nymphs) from Aurilândia and Itumbiara, state of Goiás, Brazil, were provided by Professor Ionizete Garcia da Silva. Triatomines (1^st^ to 5^th^ instars and adult insects) were kept at the Insectarium of the Institute of Biological Sciences, University of Brasília (Brazil), at 26 ± 1°C and 60 – 70% relative humidity, under 12/12h light/dark cycle for development and mating. The triatomines were periodically fed on *Gallus gallus domesticus* for approximately 30 min.

The SGs from 5^th^ instar nymphs and adults were dissected under a stereoscopic microscope at 4X magnification in sterile phosphate-buffered saline. For transcriptomic analysis, a pool of 50 SGs was transferred to a microcentrifuge tube containing 500 µL of cold TRIzol^®^ reagent (Invitrogen, EUA). The sample was stored at -80°C before total RNA extraction. For proteomic analysis, a pool of 25 SG pairs was transferred to a microcentrifuge tube containing a cocktail of protease inhibitors (Roche, USA) and gently perforated. The collected sample was centrifuged at 12,000 x *g* for 15 min, and the soluble fraction was stored at -80°C until use.

### 2.2 Total RNA Extraction, Sequencing and Bioinformatics Analysis

For transcriptomic analysis, total RNA was isolated from the samples using the TRIzol^®^ reagent following the manufacturer’s instructions. The isolated RNA was quantified using Qubit 2.0 (Invitrogen, USA). RNA sample quality was confirmed by lab-on-chip analysis using the Agilent 2100 Bioanalyzer System (Agilent Technologies, Santa Clara, CA, EUA). Finally, the sample was transferred to a RNAstable^®^ microtube (Biomatrica, San Diego, USA). cDNA library construction and sequencing were performed by Macrogen, Inc (Seoul, Korea). The cDNA library was constructed using the TruSeq^®^ stranded mRNA LT Sample Prep Kit (Illumina, San Diego, CA).

RNA-seq sequencing (paired-end read mode) was performed on Illumina NovaSeq 6000 system (Illumina, San Diego, CA, USA). The raw data were checked for data quality using FastQC (Babraham Institute, Cambridge, UK) and then trimmed using Trimmomatic (Bolger et al., 2014) to remove adapter sequences and low-quality bases. Trimmed reads were *de novo* assembled using Trinity software (Grabherr et al., 2011) and then were clustered into non-redundant transcripts using CD-HIT-EST software (Li and Godzik, 2006; Fu et al., 2012). The extracted CDS were aligned to the NR protein database of the NCBI and the Gene Ontology (GO) database (Ashburner et al., 2000) for functional annotation using Blastx tool (Madden, 2013). The tool Reverse Position-Specific BLAST (RPS-BLAST) (Altschul et al., 1997) was used to search for conserved protein domains in the Pfam (Finn et al., 2014), SMART (Ponting et al., 1999), Kog (Tatusov et al., 2003) and conserved domains database (CDD) (Marchler-Bauer et al., 2009; Lu et al., 2020). In addition, sequences were submitted to the SignalP server (Petersen et al., 2011). Finally, the contigs were automatically annotated using a program written by JMCR that searches a vocabulary of nearly 300 words found in the matches of the different databases. Additional manual annotation was done as required. The abundance of reads was reported in FPKM (Fragments Per Kilobase Million) (Mortazavi et al., 2008). CDS and their protein sequences were mapped into a hyperlinked Excel spreadsheet (**Supplementary Table 1**).

### 2.3 Multiple Alignment and Phylogenetic Analysis

Jalview software (Waterhouse et al., 2009) was used to build, edit and integrate the multiple sequence alignment analyses using MUSCLE (Edgar, 2004). The sequences used in the alignments were obtained from the NR protein database of the NCBI and are represented by six letters followed by their NCBI GI number. The letters derive from the first three letters of the genus and the first three letters of the species name. For phylogenetic analysis, selected extracted CDS from *T. sordida* SGs were compared against the NR database using the PSI-BLAST tool (Position-Specific Iterative Basic Local Alignment Search Tool) on NCBI. The most similar homolog sequences (based on the *e-value*) were selected. MEGA software (Kumar et al., 2018) was used for assembling multiple sequence alignments and infer evolutionary trees. The best model for amino acid substitution was ascertained using MEGA’s “Find Best Protein Models” tool. The trees were estimated using the following features: 1) lipocalin: maximum likelihood statistical method, WAG model of amino acid substitution and gamma distribution; 2) trialysin: maximum likelihood statistical method, WAG model of amino acid substitution and gamma distribution; 3) Kazal: maximum likelihood statistical method, WAG model of amino acid substitution and gamma distribution with invariant sites. Tree reliability was tested by the bootstrap method (N = 1000).

### 2.4 High-Performance Liquid Chromatography Coupled With Tandem Mass Spectrometry (LC-MS/MS) and Bioinformatic Analysis

The soluble fraction containing approximately 200 µg of protein was precipitated with ethanol/acetone. The sample was resuspended in 8 M urea in 20 mM triethylammonium bicarbonate, reduced, trypsin digested and desalted as described (Santiago et al., 2018). Desalted peptides were dried, suspended in 0.1% formic acid in water, quantified by Qubit^®^ and adjusted at a concentration of 0.5 µg/µL. Two replicates of 2.5 µg tryptic peptides were then subjected to LC-MS/MS analysis. Each peptide sample was loaded by an autosampler into the trap column at a flow rate of 4 µl.min−1 in 98% buffer A (0.1% formic acid in water) and 2% buffer B (0.1% formic acid in acetonitrile 80%). The peptides were separated using a Dionex UltiMate™ 3000 RSLCnano system capillary column (Thermo Fisher Scientific, Waltham, MA, USA) at a constant flow 230 nL/min in a 20 cm analytical column (75 µm inner diameter) packed with 3 µm C18 beads (Reprosil Pur-AQ, Dr. Maisch, Germany) with a 50 min gradient ranging for in-gel protein digestion samples and a 190 min gradient ranging from 5 to 35% acetonitrile in 0.1% formic acid, and directly loaded into Orbitrap Elite™ hybrid ion trap-orbitrap mass spectrometer (Thermo Fisher Scientific, San Jose, CA, USA) under electrospray ionization. For proteomic analysis, the PatternLab for Proteomics 4.0 software (Carvalho et al., 2016) was used. The.raw files were subjected to search against the *T. sordida* transcriptome databank using the following parameters: enzymatic specificity in protein hydrolysis as semi-tryptic with tolerance for up two lost cleavages; fixed modification of carboxymethylated cysteine residues; fragment bin tolerance of 0.02; fragment bin offset of 0.0; MS/MS and precursor tolerance of 40 ppm; mass range search between 550 and 5,500 Da. Search results were post-processed and statistically filtered *via* Search Engine Processor (SEpro). The result of Peptide Sequence Matches was obtained from this statistical processing that took into account only the False Discovery Rate (FDR) peptides of each sample. The program automatically calculated FDR values by defining as acceptable only FDR < 2% for peptides and FDR < 1% for proteins. For each protein group, Primary Score, Secondary Score, and DeltaCN score values were used to generate a Bayesian discriminator function automatically.

### 2.5 Statistical Method Applied in the Correlation Analysis

The degree of linear association between reads (FPKM) and proteins (spectrum count) abundance was measured by calculating the coefficient correlation of Pearson using GraphPad Prism version 8.00 for Apple, GraphPad Software, San Diego California USA, www.graphpad.com software. To estimate the strength of the relationship between data, P value (derived from t test) was used.

### 2.6 Data Access

The raw data were deposited in the National Center for Biotechnology Information (NCBI) under Bioproject PRJNA749011, Biosample SAMN20349839 and Sequence Read Archive (SRA) accession SRR15218234. The assembled coding sequences and their peptide translations were submitted to DDBJ/EMBL/GenBank *via* the Transcriptome Shotgun Assembly (TSA) project under the accession GJHW00000000. The version described in this paper is the first version, GJHW01000000.

The mass spectrometry proteomics data have been deposited to the ProteomeXchange Consortium *via* the PRIDE (Perez-Riverol et al., 2019) partner repository with the dataset identifier PXD028350.

## 3 Results

### *3.1 De Novo* Assembly of *Triatoma sordida* Salivary Gland Transcripts

The high-throughput sequencing of the *T. sordida* salivary gland cDNA library yielded 57,645,372 high quality reads assembled into 26,670 CDS. Based on sequence homology, a set of 16,683 transcripts, corresponding to 62.6% of the generated CDS, were extracted from the functional annotation analysis (**Table 1**). The CDS were classified into seven different categories: Housekeeping (H), salivary putative Secreted (S), Immunity related (IR), Unknowns (U), Proteasome machinery (P), Transposable elements (TE) and Viral product (Vr). The FPKM values of the CDS from derived categories were clustered. Remarkably, it is possible to observe a high abundance of reads from the S category, comprising 79.5% of the total FPKM (including 77.5% of the reads and 735 CDS). The H category comprehends 12.1% of the FPKM (distributed in 18.3% of the reads and 6,325 CDS). Together, these two categories covered over 91% of the FPKM from functionally annotated transcripts. Because no functional assignments against any database could be made, 8,092 CDS were classified within the U category, representing 7.6% of the total FPKM and 3.0% of reads. P (FPKM = 0.6%), IR (FPKM = 0.1%), TE (FPKM < 0.1%) and Vr (FPKM < 0.1%) categories accounted for less than 0.8% of the FPKM from functionally annotated CDS. The H and U categories showed a large repertoire of CDS (6,325 and 8,092, respectively), a diversity that may reflect their essential functions regarding cellular maintenance. Functional classification and expression levels of the transcripts extracted from *T. sordida* sialotranscriptome are condensed in **Table 1**.

**Table 1:** Functional classification and expression levels of the coding sequences (CDS) extracted from *Triatoma sordida* sialotranscriptome.

Category	Number of CDS	Number of reads	%reads	Average number of reads/CDS	FPKM	%FPKM
**Putative Secreted**	735	9,340,897	77.5	12,709	2,714,435	79.5
**Housekeeping**	6,325	2,200,836	18.3	348	414,372	12.1
**Unknowns**	8,092	365,839	3.0	45	260,915	7.6
**Proteasome machinery**	501	102,188	0.9	204	18,925	0.6
**Immunity related**	126	24,319	0.2	193	4,857	0.1
**Transposable element**	894	13,458	0.1	15	2,551	<0.1
**Viral product**	10	2,474	<0.1	247	45	<0.1
**Total**	**16,683**	**12,050,013**	**100**		**3,416,099**	**100**

The values in bold correspond to the total for each class, group or subgroup.

### 3.2 The Housekeeping Category

Based on their possible functions, CDS from the H category were further distributed into 21 groups (**Table 2**). Protein synthesis machinery revealed to be the most abundant group, corresponding to 37.1% of the total FPKM value, followed by: Protein modification (FPKM = 9.3%), Energy metabolism (FPKM = 7.8%), Oxidant metabolism/detoxification (FPKM = 6.7%), Signal transduction (FPKM = 6.0%), Extracellular matrix (FPKM = 5.7%), and Transporters and channels (FPKM = 5.7%). Accordingly, gene transcription and translation groups are enriched in tissues with a secretory role, in this case, into the salivary lumen, reflecting their essential role in blood acquisition performance during feeding. Altogether, the remaining groups from the H category are represented by a FPKM below 22%.

**Table 2:** Functional classification and expression levels of the CDS attributed to the Housekeeping category extracted from *Triatoma sordida* sialotranscriptome.

Housekeeping Category	Number of CDS	Number of reads	%reads	Average number of reads/CDS	FPKM	%FPKM
**Protein synthesis machinery**	462	533,374	24.2	1,154	153,782	37.1
**Protein modification**	359	365,123	16.6	1,017	38,371	9.3
**Energy metabolism**	386	158,957	7.2	412	32,260	7.8
**Oxidant metabolism/Detoxification**	101	72,003	3.3	713	27,895	6.7
**Signal transduction**	1,562	179,296	8.2	115	24.835	6.0
**Extracellular matrix**	171	43,294	2.0	253	23,761	5.7
**Transporters and channels**	415	159,587	7.3	385	23,567	5.7
**Protein export**	432	101,734	4.6	235	18,456	4.5
**Transcription machinery**	773	152,434	6.9	197	17,459	4.2
**Lipid metabolism**	268	89,681	4.1	335	11,836	2.9
**GPI anchored extracellular or exosomal**	30	22,510	1.0	750	8,704	2.1
**Cytoskeletal proteins**	304	56,943	2.6	187	7,414	1.8
**Nuclear regulation**	299	28,771	1.3	96	4,904	1.2
**Carbohydrate metabolism**	166	48,878	2.2	294	4,459	1.0
**Amino acid metabolism**	140	45,558	2.1	325	4,375	1.1
**Storage**	26	60,704	2.8	2,334	3,078	<0.1
**Nucleotide metabolism**	144	17,692	0.8	123	2,820	<0.1
**Transcription factor**	166	20,953	1.0	126	2,530	<0.1
**Detoxification**	43	18,567	0.8	431	1,842	<0.1
**Intermediary metabolism**	49	21,082	1.0	430	1,750	<0.1
**Nuclear export**	29	3,697	0.2	127	275	<0.1
**Total**	**6,325**	**2,200,836**	**100**		**414,372**	**100**

The values in bold correspond to the total for each class, group or subgroup.

### 3.3 The Secreted Category

The CDS attributed to the S category expressed in *T. sordida* SGs were divided into nine main groups, and these were further subdivided into 27 subgroups (**Table 3**). Lipocalin is the most abundant protein family, corresponding to 45.1% of the FPKM within the S category (48.8% reads and 136 CDS). A pallidipin (Hem-c11226_g1_i1.p1), followed by four different salivary lipocalins (Hem-c15348_g11_i1.p1, Hem-c15366_g5_i2.p1, Hem-c15366_g5_i3.p1 and Hem-c13513_g1_i1.p1), with FPKM values corresponding to 4.2%, 3.5%, 2.4%, 2.4% and 2.1%, respectively, were the most abundant lipocalin contigs. The remaining reads from the S category were divided as follows: Hemiptera specific families (FPKM = 32.0%), Protease inhibitors (FPKM = 12.9%), Enzymes (FPKM = 3.8%), Ubiquitous protein families (FPKM = 3.1%), and Other salivary secreted proteins (FPKM = 3.1%). The Pheromone binding protein, Odorant binding protein and Juvenile hormone-binding protein groups, encompass 0.1% of the total FPKM (**Table 3**). Two subgroups, short trialysin/trialysin from Hemiptera specific families and Kazal-type from protease inhibitors, are highlighted as they alone accounted for 30.3% and 12.8% of the FPKM, respectively. Interestingly, together with lipocalins, trialysin and Kazal-type are the major putative secreted protein families from *T. sordida* sialotranscriptome, comprising more than 88% of the FPKM directly related to the S category. **Table 3** depicts in more detail the distribution of the extracted secreted CDS and their respective abundances.

**Table 3:** Functional classification and expression levels of the CDS attributed to the putative Secreted category extracted from *Triatoma sordida* sialotranscriptome.

Putative Secreted Category	Number of CDS	Number of reads	%reads	Average number of reads/CDS	FPKM	%FPKM
**Lipocalin**						
Triabin	89	3,180,268	34.1	35,733	936,123	34.5
Pallidipin	21	1,281,977	13.7	61,047	255,970	9.4
Other lipocalin	18	61,350	0.7	3,408	19,885	0.7
Triatin	5	32,225	0.3	6,445	11,353	0.4
Procalin	3	1,184	<0.1	395	1,352	0.1
Total	136	4,557,004	48.8		1,224,683	45.1
**Hemiptera specific families**						
Short trialysin/trialysin	10	2,763,272	29.6	276,327	821,268	30.3
Salivary protein MYS precursor/Hemolysin like	17	217,213	2.3	12,777	45,652	1.7
Triatoma and Panstrongylus specific salivary proteins 16kDa	3	1,123	<0.1	374	290	<0.1
Tsor 8kDa basic salivary peptide	6	157	<0.1	26	150	<0.1
Cisteine rich secreted protein	4	16	<0.1	4	19	<0.1
Total	40	2,981,781	32.0		867,380	32.0
**Protease inhibitors**						
Kazal-type	27	229,814	2.5	8,512	347,414	12.8
Cystatin	3	1,357	<0.1	452	844	<0.1
Serpin	6	7,385	<0.1	1,231	618	<0.1
Pacifastin	6	1,435.	<0.1	239	250	<0.1
Mucins	20	279	<0.1	14	383	<0.1
Kunitz-type	7	208	<0.1	30	13	<0.1
Total	69	240,478	2.6		349,522	12.9
**Enzymes**						
Inositol phosphate phosphatase	14	390,510	4.2	27,894	54,472	2.0
Salivary Trypsin	8	232,304	2.5	29,038	31,074	1.1
Metalloproteases	5	78,930	0.8	15,786	10,240	0.4
79kDa salivary apyrase	5	30,899	0.3	6,180	7,242	0.3
Nucleases	4	2,237	<0.1	559	200	<0.1
Secreted caboxylesterase	8	1,841	<0.1	230	106	<0.1
Lysosomal aspartic protease	2	888	<0.1	444	75	<0.1
Lipase	5	420	<0.1	84	58	<0.1
Total	51	738,028	7.9		103,467	3.8
**Ubiquitous protein families**						
Antigen 5	13	438,036	4.7	33,695	77,434	2.9
Conserved insect secreted protein	13	15,438	0.1	1,188	6,212	0.2
Low density lipoprotein receptor	5	16	<0.1	3	18	<0.1
Total	31	453,490	4.6		83,664	3.1
**Other salivary secreted protein**	362	361,649	3.9	999	82,797	3.1
**Pheromone binding protein**	9	4,918	<0.1	546	2,145	<0.1
**Odorant binding protein**	25	1,608	<0.1	64	470	<0.1
**Juvenile hormone binding protein**	12	1,940	<0.1	162	307	<0.1
**Total**	**735**	**9,340,897**	**100**		**2,714,435**	**100**

The values in bold correspond to the total for each class, group or subgroup.

### 3.4 *Triatoma sordida* Sialoproteome

*T. sordida* salivary soluble extract was subjected to LC/MS-MS mass spectrometry to identify the protein products encoded by its SGs through large-scale proteomic analysis, yielding a total of 1,571 spectrum counts distributed among 132 identified proteins (**Supplementary Table 2**), observed at both protein and mRNA level. The amount of the result found here is comparable to other previously reported Triatominae sialoproteomes, which in general are far from the number of contigs extracted from the SG transcriptomic analysis (Santiago et al., 2020a).

**Figure 1:**
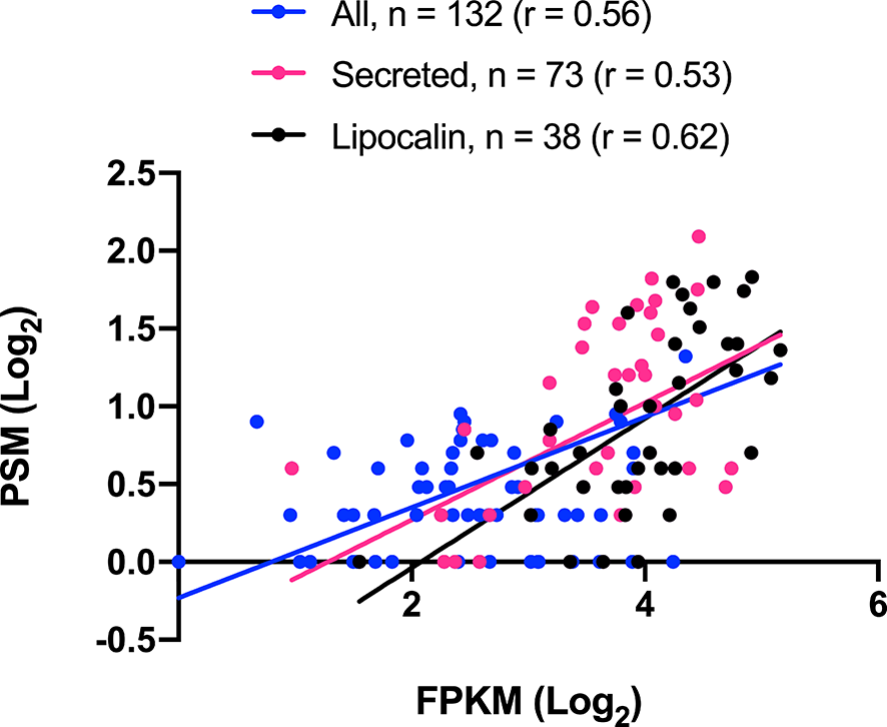
*Triatoma sordida* salivary transcriptome/proteome correlation. The scatterplot illustrates the Pearson’s correlation (*r.*) between CDS (FPKM) and proteins (spectrum count) from *T. sordida* salivary gland transcriptome and salivary gland extract proteome. It shows a statistically significant P value (p<0.0001) in the three groups tested: All identified proteins (blue), Secreted identified proteins (pink), and Lipocalin identified proteins (black).

A summary of all identified proteins according to their putative function is shown in **Table 4**. It is worth highlighting the 73 identified proteins belonging to the S category. Lipocalin, Enzymes, Hemiptera specific families, Ubiquitous protein families, Other salivary secreted protein, Protease inhibitors and Odorant binding protein match, respectively, to 42.0%, 22.5%, 10.1%, 5.9%, 4.9%, 1.4% and < 0.1% of the total spectrum count. Aiming to explore a quantitative relationship between mRNA and protein levels, a correlation (r) analysis was performed, revealing a moderate positive correlation coefficient among all identified proteins (r = 0.6), identified proteins from the S category (r = 0.5) and identified lipocalins (r = 0.6) (**Figure 1**). An explanation for these values could be that several proteins may be secreted during salivation following feeding stimulation. In addition, it has been suggested the rate of gene expression is influenced not exclusively by transcription and translation but also by mRNA and protein turnover (Li and Biggin, 2015; Shieh, 2019).

**Table 4:** Classification and abundance of proteins identified in the salivary glands of *Triatoma sordida* following LC-MS/MS analysis.

Protein identification	Number of proteins	Spectrum count	% Spectrum count
***Secreted***			
**Lipocalin**			
Triabin	31	532	33.9
Pallidipin	5	113	7.2
Other lipocalins	1	10	0.6
Triatin	1	5	0.3
Total	38	660	42.0
**Enzymes**			
Inositol phosphaye phosphatse	8	252	16.0
Salivary trypsin	4	86	5.5
79kDa salivary apyrase	2	16	1.0
Total	14	354	22.5
**Hemiptera specific familiesfamilies**			
Short trialysin/Trialysin	2	133	8.5
Salivary protein MYS precursor/Hemolysin like	2	19	1.2
Triatoma and Panstrongylus specific salivary protein of 16kDa	1	7	0.5
Total	5	159	10.1
**Ubiquitous protein families**			
Antigen 5	4	92	5.9
**Other salivary secreted protein**	7	77	4.9
**Protease inhibitors**			
Kazal type	4	22	1.4
**Odorant binding protein**	1	1	<0.1
**Total**	**73**	**1372**	**86.9**
***Immunity***	**2**	**7**	**0.5**
***Housekeeping***			
**Protein modification**	7	44	2.8
**Cytoskeletal protein**	7	25	1.6
**Energy metabolism**	5	12	0.8
**Protein synthesis machinery**	4	11	0.7
**Signal transduction**	5	11	0.7
**Lipid metabolism**	3	10	0.6
**Storage**	3	9	0.6
**Extracellular matrix**	1	8	0.5
**Oxidant metabolism/Detoxification**	3	6	0.4
**Amino acid metabolism**	1	6	0.4
**Detoxification**	1	3	0.2
**Transporters and channels**	1	2	0.1
**Transcription machinery**	1	2	0.1
**Carbohydrate metabolism**	1	2	0.1
**Protein export**	1	1	<0.1
**Total**	**44**	**152**	**9.7**
***Unknown***	**13**	**47**	**3.0**
**Total**	**132**	**1571**	**100**

The values in bold correspond to the total for each class, group or subgroup.

## 4 Discussion

### 4.1 Functional Analysis of *Triatoma sordida* Sialome

In this study, we explored the sialotranscriptome and sialoproteome of *T. sordida* triatomine. The most striking protein families of the analysis are described in detail in the following section, highlighting their putative salivary function within their hematophagy context.

#### 4.1.1 Lipocalin Family

*T. sordida* sialotranscriptome analysis revealed lipocalins as the most expressed gene family within the putative S category. Indeed, a common feature of Triatominae sialotranscriptomes reported so far is the abundance of lipocalin secreted transcripts (Santiago et al., 2020a). In line with the sialotranscriptome, the lipocalins were the most abundant proteins identified in the sialoproteome analysis, indicating synchronicity between transcript and peptide expressions (**Figure 1**). Lipocalins comprise a large group of small extracellular proteins belonging to the calicin superfamily, which has been described in all Triatominae sialotranscriptomes previously reported (Santiago et al., 2020a). The lipocalin structure is comprised of a conserved three-dimensional structure of a single, eight-stranded hydrogen-bonded antiparallel β-barrel, folded to form a binding pocket at its centre that encloses an internal ligand-binding site (Flower et al., 2000). The pockets present a wide variety of shape, hydrophobicity, and charge characteristics, making lipocalin members heterogeneous. Lipocalins usually bind to small hydrophobic molecules, cell surface receptors or other proteins, targeting different points of the host’s hemostatic system (Hernández-Vargas et al., 2016).

**Figure 2:**
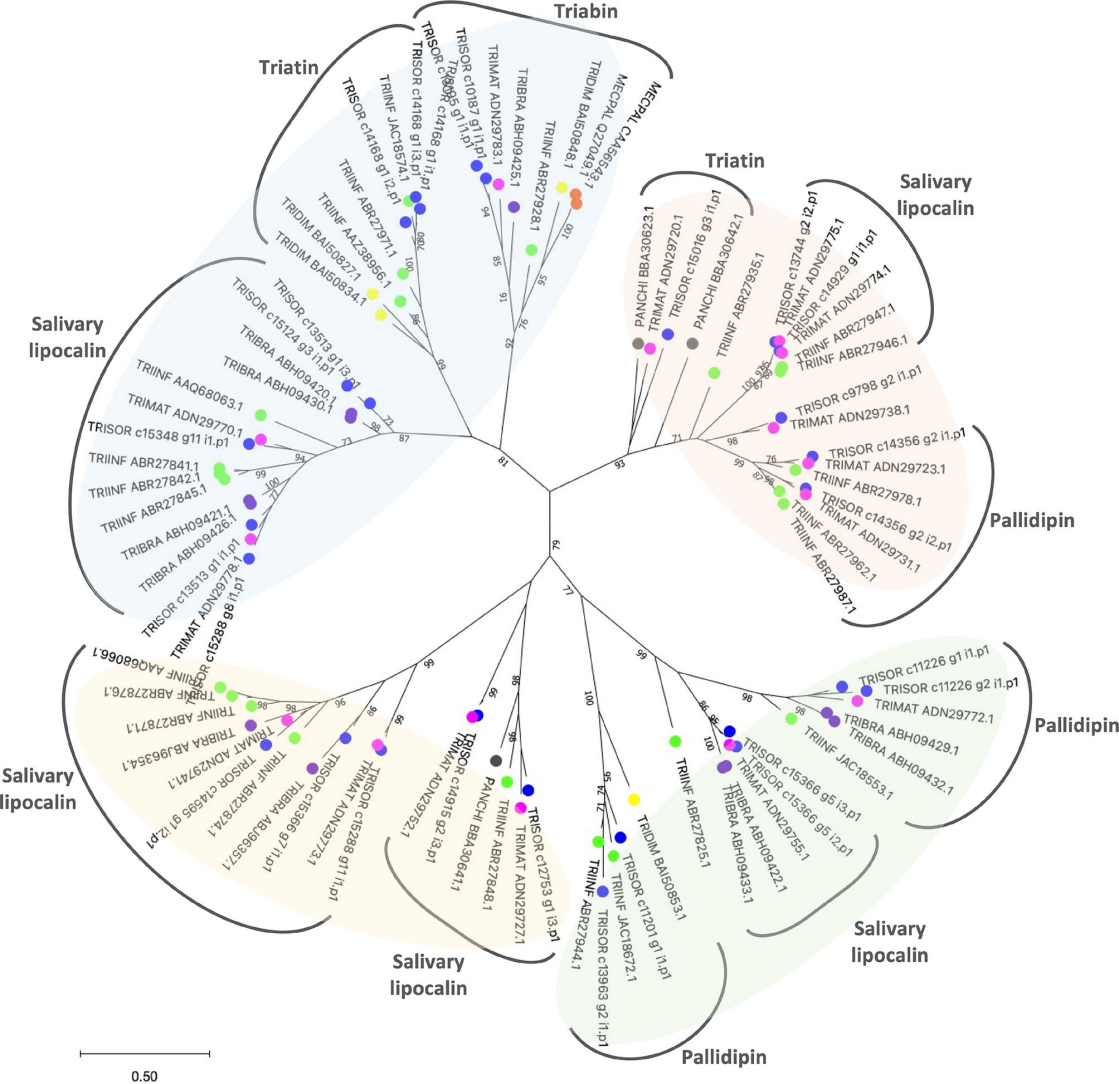
Maximum likelihood tree with *Triatoma sordida* salivary lipocalins. The distance tree among lipocalin sequences is derived from the alignment of *T. sordi*da CDS extracted from the sialotranscriptome analysis and other lipocalin sequences from Triatominae. The amino acid sequences are identified as described in the *Methods* section. The coloured circles identify the species whose sequences were used: blue, *T. sordida*; magenta, *Triatoma matogrossensis*; purple, *Triatoma brasiliensis*; green, *Triatoma infestans*; yellow, *Triatoma dimidiata*; grey, *Panstrongylus chinai*. The numbers at the nodes indicate the bootstrap values. The scale on the bottom measure evolutionary distance in substitutions per amino acid.

To estimate the relationships among *T. sordida* lipocalin sequences, a phylogenetic tree was constructed (**Figure 2**). The result shows lipocalin CDS grouped within four main clades, including different lipocalins such as pallidipin, triatin, triabin and other salivary lipocalin members, repeatedly in the branches. This indicates the strong similarities among members within the family. As previously reported in other triatomine species (Santiago et al., 2020a), sialotranscriptomic and phylogenetic analysis suggests the salivary lipocalins from *T. sordida* evolved through gene duplication and divergence, giving rise to an abundant salivary lipocalin multigene family. In addition, the end of the branch patterns suggests *T. sordida* is closely related to *Triatoma matogrossensis* species.

The collagen exposed *via* wounding of the skin due to insect bite leads to the activation of platelets, which secret thromboxane A_2_ (TXA_2_) and release mediators of platelet activation and inflammation, such as ADP and serotonin, potentiating the activation response (Broos et al., 2011). Salivary proteins from blood-feeding arthropods bind to collagen, ADP and TXA_2_ to quickly reduce the local free concentrations of these agonists (Alvarenga et al., 2010). The pallidipin lipocalin inhibits collagen-mediated aggregation of platelets while it does not attenuate platelet aggregation by other agonists, including ADP and thrombin, suggesting the protein is a TXA_2_ scavenger (Noeske-Jungblut et al., 1995; Assumpção et al., 2010). The triatin lipocalin has been found in different triatomine sialotranscriptomes. However, its molecular role is still unknown.

Although triabins were initially classified as lipocalins, due to their 3D fold, they have been placed in a distinct family strongly related to lipocalins within the superfamily of calicins (Flower et al., 2000). Triabin inhibits thrombin-induced platelet aggregation by interacting with thrombin exclusively *via* its fibrinogen-recognition exosite (Noeske-Jungblut et al., 1995; Fuentes-Prior et al., 1997). Since triabin isolation from the saliva of *Triatoma pallidipennis*, its sequence has been identified in several Triatominae sialotranscriptomes (Sant’Anna et al., 2002; Ribeiro et al., 2004; Santos et al., 2007; Francischetti et al., 2009; Andersen, 2010; Bussacos et al., 2011; Ribeiro et al., 2012a; Santiago et al., 2016; Hernández-Vargas et al., 2017; Santiago et al., 2018). The lineage-specific expansion of lipocalins in Triatominae is reflected in the protein abundances and the redundancy of fundamental roles. High protein concentrations are essential to guarantee blood-feeding success, such as targeting agonists that elicit a rapid host’s hemostatic response. Furthermore, the variety of proteins coupled with differential expression may also be a strategy to evade the host’s immune responses.

#### 4.1.2 Hemiptera Specific Protein Families

*T. sordida* sialotranscriptome analysis revealed 40 CDS coding for Hemiptera specific families (FPKM = 32%), including short trialysin/trialysin, MYS/hemolysin, *Triatoma* and *Panstrongylus* specific 16 kDa salivary protein, Tsord 8 kDa basic salivary peptide and cysteine-rich secreted protein (**Table 3**). PSI-BLAST searches revealed these extracted transcripts match with Hemiptera protein sequences that are not similar to any other in the NR database. Along with new transcriptome studies, such molecules should be reported in other species’ salivary/venom content.

Trialysin is a pore-forming lytic protein from *Triatoma infestans* saliva, permeating and lysing different cell types, such as bacterial and mammalian cells (Amino et al., 2002). The _˜_ 22 kDa mature form and the _˜_ 6 kDa short form of trialysins were already described (Ribeiro et al., 2012b). The molecule is synthesized and stored as a precursor in the SGs. It is processed by limited proteolysis after salivary release, a mechanism to avoid self-damage during its synthesis and secretion (Martins et al., 2008). Interestingly, trialysins seem to be restricted to some triatomine species, and their members have been found in *T. infestans* and *T. matogrossensis* sialotranscriptomes (Ribeiro et al., 2012b). It was suggested that triapsin, a serine protease from *T. infestans* saliva, may be responsible for trialysin activation (Amino et al., 2002). However, no triapsin sequence was found here in the *T. sordida* sialotranscriptome.

The venoms of the bugs *Pristhesancus plagipennis, Platymeris biguttatus* and *Psytalla horrida* (Hemiptera: Reduviidae) are made up of a complex mixture of proteins including a cytolytic toxin homologous to the *T. infestans* tryalisin, named redulysin (Walker et al., 2017; Fischer et al., 2020). PSI-BLAST searches with trialysin sequences as a query retrieved only 19 trialysin protein sequences from *T. matogrossensis, T. infestans, Panstrongylus megistus* and the Hemiptera bean bug *Riptortus pedestris*, and 21 redulysins from *Platymeris rhadamanthus* and *P. plagipennis* (accessed on April 15). In Hemiptera venom, the cytolytic activity of redulysins may contribute to the liquefaction of the prey tissue in a way to facilitate feeding through the narrow channel of the proboscis in this invertebrate predator (Walker et al., 2017). Although the lytic potential of trialysin is well experimentally established in triatomines, its biological role in saliva remains unclear. It has been suggested it might have an antimicrobial function, controlling the growth of microorganisms in saliva or even interfering with the host’s immune cell response (Amino et al., 2002).

The lytic properties of synthetic peptides encompassing portions of the Lys-rich N-terminus motif of trialysin were tested. It was shown they efficiently induce the lysis of *T. cruzi* and *Escherichia coli* membranes, an activity that is less effective against human erythrocytes (Amino et al., 2002; Martins et al., 2006). The study indicates this conserved region fold into an amphipathic α-helix conformation, a structure predicted to exert antimicrobial/lytic activity. It was proposed that while trialysin amphipathicity is essential for bacterial lysis, an increase in hydrophobicity is correlated with the lysis of erythrocytes (Chen et al., 2005). Furthermore, the structural details involving the N- and C- termini of the trialysin members and the lipid composition of the target cell membrane are directly linked to the differences in selectivity and efficiency of the synthetic peptides (Martins et al., 2006).

Here, ten short trialysin/trialysin CDS, corresponding to 30.3% of total FPKM from the S category were revealed (**Table 3**), four of them are abundantly expressed as shown by their individual FPKM values (Hem-c_15059_g2_i1.p1 = 9.4%, Hem-c_10816_g1_i1.p1 = 7.8%; Hem-c_15059_g1_i2.p2 = 3.5%; Hem-c_14763_g9_i1.p2 = 1.9%). Accordingly, sialoproteome analysis results detected one trialysin protein (Hem-c_15366_g14_i1.p1), which corresponds to 7.8% of the total spectrum count. As far as we know, the abundant feature of secreted trialysin in the saliva of a triatomine species was previously reported in *T. infestans* (Assumpção et al., 2008; Schwarz et al., 2014) and *T. matogrossensis* saliva (Assumpção et al., 2012).

**Figure 3:**
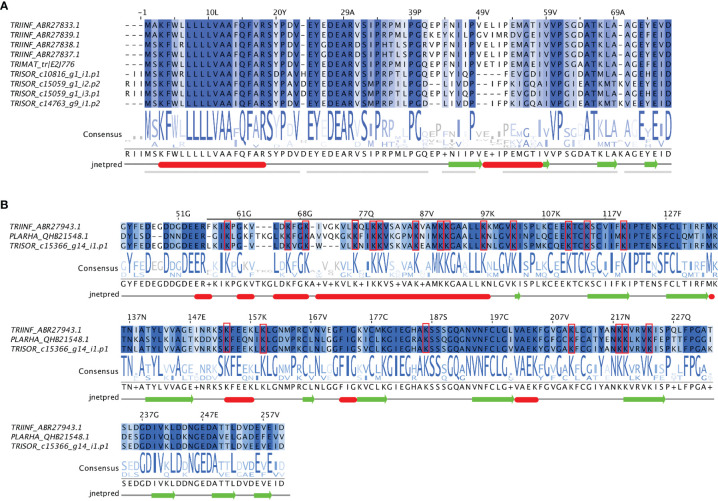
The trialysins from *Triatoma sordida* sialotranscriptome. **(A)** Multiple sequence alignment of short-trialysin members extracted from *T. sordida* sialotranscriptome (TRISOR_c10816_g1_i1.p1; TRISOR_c15059_g1_i2.p2; TRISOR_c15059_g1_i3.p1; TRISOR_c14763_g9_i1.p2) and other homologous short-trialysin sequences from *Triatoma infestans* (TRIINF_ABR27833.1; TRIINF_ABR27839.1; TRIINF_ABR27838.1; TRIINF_ABR27837.1) and *Triatoma matogrossensis* (TRIMAT_tr/E2J776) **(B)** Multiple sequence alignment of a r-trialysin member extracted from *T. sordida* sialotranscriptome (TRISOR-c15366_g14_i1.p1) and two homologous members, a r-trialyisn from *Triatoma infestans* (TRIINF_ABR27943.1) and a redulysin from *P. rhadamanthus* (PLARHA_QHB21548.1). The sequences are identified as described in *Methods* section. The alignments indicate conserved residues in a blue scale background. The grey bar marks the consensus motif of trialysins, red rectangles mark Lys residues. Consensus logo sequence shows the frequency of amino acids in the multiple sequence alignment. The height of symbols within the stack indicates the relative frequency of each amino acid at that position. Secondary structure predictions were inferred using JNet Secondary Structure Prediction. The sequence for which the prediction was made is the first one in the alignment.

Multiple sequence alignment reveals that the short trialysins from *T. sordida, T. infestans* and *T. matogrossensis* are strongly conserved, as well as the r-trialysins from *T. sordida, T. infestans* and *P. rhadamanthus* (**Figure 3A**). Moreover, the secondary structure prediction pattern indicates the presence of α-helical structures in the N-terminus of short trialysins and r-trialysins. It is worth noting that the short trialysins are not Lys-rich and are homologous to the most N-terminal region of *T. infestans* r-trialysin (not shown in these alignments). *T. sordida* r-trialysin sequence has been shown to be conserved in comparison with *T. infestans* and *P. rhadamanthus*, possessing the motif predicted to have the lytic property (indicated with a grey bar above the alignment, **Figure 3B**). These shared patterns point to a common structural fold and biological function among these related trialysins. We performed a Kyte and Doolittle hydrophobicity analysis, which indicated that the short-trialysin Hem-c_15059_g1_i2.p2 presents a highly hydrophobic region in the N-terminus and, to a lesser extent, also in the C-terminus. At the same time, the plot shape of the r-trialysin Hem-c_15366_g14_i1.p1 shows many hydrophobic and hydrophilic stretches (**Supplementary Figure 1**). The amino acids contributing to hydrophobicity seems to be part of the predicted α-helices and may fold in membrane-spanning domains.

**Figure 4:**
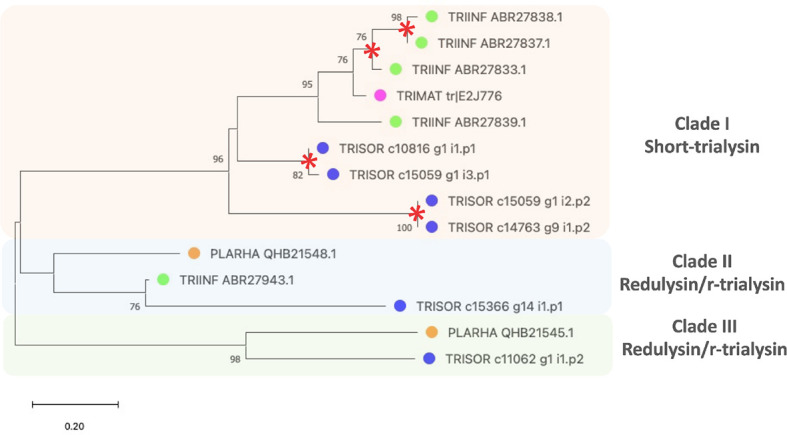
The phylogenetic tree of trialysins from *Triatoma sordida* sialotranscriptome. The distance tree among trialysin sequences is derived from the alignment of *T. sordida* CDS extracted from the sialotranscriptome analysis and other trialysin sequences from Hemiptera (extracted from the non-redundant protein database of the NCBI applying PSI-BLAST algorithm) identified as described in *Methods* section. The colored circles identify the species whose sequences were used: blue, *T. sordida*; magenta, *Triatoma matogrossensis*; green, *Triatoma infestans*; orange, *P. rhadamanthus.* Red asterisks indicate gene duplication events. The number at the nodes shows the bootstrap values. The scale on the bottom measure evolutionary distance in substitutions per amino acid.

To find the evolutionary relationships among trialysin/redulysin members, a phylogenetic tree was constructed (**Figure 4**). The result shows *T. infestans, T. matogrossensis* and *T. sordida* short trialysins to be monophyletic, grouping together in Clade I. Gene duplication events (red asterisk) occurred, resulting in short-trialysin homologs in *T. infestans* and *T. sordida* (**Figure 4**). In addition, it is possible to infer that Clade I and Clade II are orthologous to Clade III, suggesting that short-trialysin and r-trialysin were recruited into saliva/venom before the split of triatomines and assassin bugs.

##### 4.1.2.1 Hemolysin-Like Secreted Salivary Protein

Cytolytic toxins are found in several bacterial genera that can permeate a wide range of target cells through pore formation, causing cell damage (Welch, 1991; Welch, 2001). As a mechanism of iron acquisition, some of these bacterial toxins are capable of lysing erythrocytes and thus are named hemolysin (Rowe and Welch, 1994). Homologs of these cytolytic molecules are constituents of arthropod venoms, and a defensive role against a threat or a protective role against bacterial infections has been suggested (Rowe and Welch, 1994; Walker et al., 2018). Hemolysin-like proteins are abundantly found in the venom gland of the assassin bug *P. plagipennis*, a venomous reduviid predator (Insecta: Hemiptera) (Walker et al., 2018). The hemolysins are also an important constituent in the venoms of *P. biguttatus* and *P. horrida* (Fischer et al., 2020). In triatomines, MYS/hemolysin members were first described in *Rhodnius prolixus* sialotranscriptome and since then, have been commonly found in Triatominae sialotranscriptomes (Ribeiro et al., 2004; Santiago et al., 2020a). *T. sordida* sialotranscriptome encompasses 17 contigs of the MYS/Hemolysin, corresponding to 1.7% of the FPKM from S category (**Table 3**). A PSI-BLAST search with *T. sordida* MYS/hemolysins as query against NR-database retrieved MYS/hemolysin sequences from *T. infestans, R. prolixus, Panstrongylus chinai, Triatoma dimidiata* and from the hemipterans *P. plagipennis* and *P. rhadamanthus*.

A key event in blood digestion by hematophagous insects is the lysis of erythrocytes, although, the hemolytic activity in saliva has not been functionally characterized. Still, since much of the protein resource is locked in the blood cells, it appears reasonable that hematophagous insects require a MYS/hemolysin molecule to open the erythrocytes before digestion of the meal products in the midgut. A hypothesis for its role is that the protein would be involved in the initial stages of the blood digestion processes, causing erythrocyte lysis in saliva and releasing its contents for further processing by digestive proteolytic enzymes (Assumpção et al., 2008).

#### 4.1.3 Protease Inhibitors

Protease inhibitors are widely distributed among living organisms, playing crucial roles in different biological processes. In triatomines, the anti-protease activity may take place in the extracellular space, namely in the salivary content or in the intracellular environment. Serine proteinase inhibitors are a frequent component of sialomes from blood-feeding arthropods, including triatomines (Tanaka-Azevedo et al., 2010; Santiago et al., 2017). Here, 69 transcripts coding for protease inhibitors were extracted, including Kazal-type, cystatin, serpin, pacifastin, mucin and Kunitz-type (**Table 3**).

The Kazal-type proteins are serine protease inhibitors from the I1 family widely found in vertebrate and invertebrate animals (Rimphanitchayakit and Tassanakajon, 2010). Invertebrate Kazal-type proteinase inhibitors may have single or multiple domains containing six conserved cysteine residues forming three intra-domain disulphide cross-links and are considered non-classic according to the numbers of amino acid residues between their half-cystines (Rimphanitchayakit and Tassanakajon, 2010). Rhodniin, dipetalogastin, infestin and brasiliensin are Kazal-type thrombin inhibitors reported in the intestines of *R. prolixus, Dipetalogaster maxima, T. infestans* and *Triatoma brasiliensis* species (Friedrich et al., 1993; Campos et al., 2002; Mende et al., 2004; Araujo et al., 2007; Lovato et al., 2011). As triatomines ingest large blood meals to ensure blood supply, an anti-clotting activity in the intestines seems to be fundamental to avoid increased blood viscosity in the digestive tract during blood acquisition and storage, thereby facilitating digestion (Araujo et al., 2007). Members of the Kazal-type family have already been reported in the saliva from hematophagous animals such as tabanids, mosquitoes, leeches and triatomines (Campos et al., 2002; Lovato et al., 2006; Rimphanitchayakit and Tassanakajon, 2010). Vasotab is a Kazal-type member from the saliva of *Hybomitra bimaculata* (Diptera: Tabanidae) that shows a vasoactive biological function (Takác et al., 2006). In addition, they are also one of the main components of the venom from hemipteran bugs (Walker et al., 2019), suggesting these protease inhibitors derive from an ancestral lineage.

**Figure 5:**
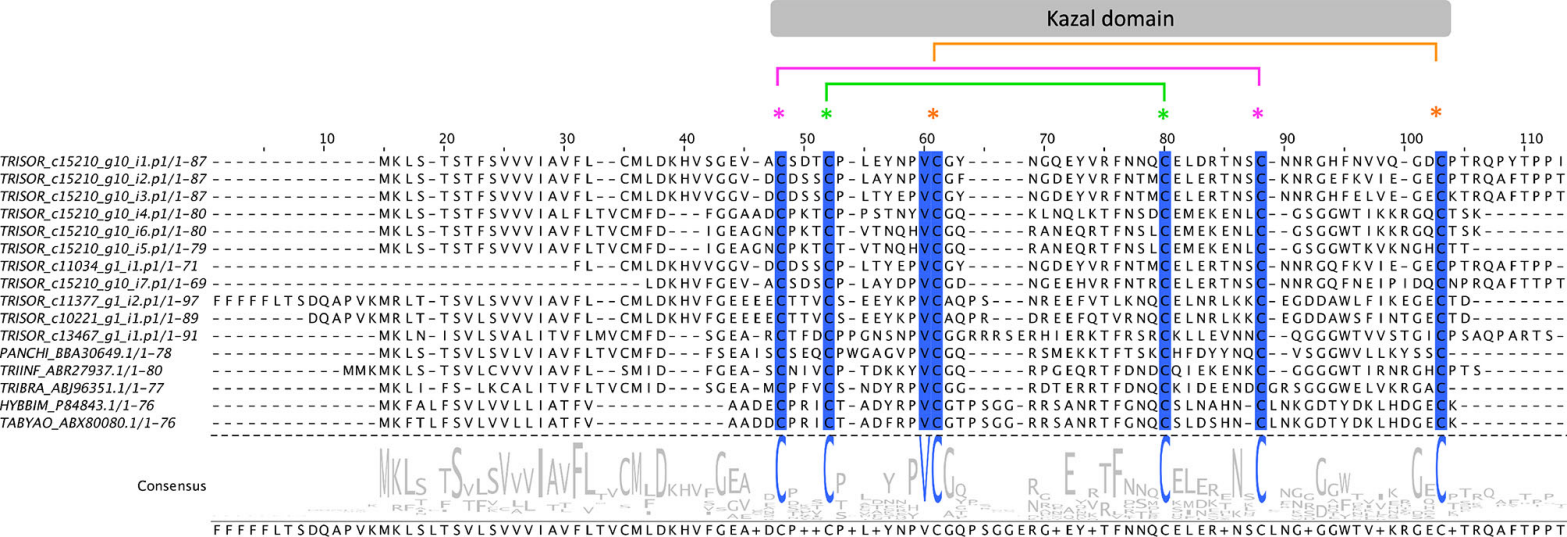
The Kazal-type members from *Triatoma sordida*. Multiple sequence alignment of Kazal-type members extracted from *T. sordida* sialotranscriptome (TRISOR_ c15210_g10_i1.p1; TRISOR_ c15210_g10_i2.p1; TRISOR_ c15210_g10_i3.p1; TRISOR_ c15210_g10_i4.p1; TRISOR_ c15210_g10_i6.p1; TRISOR_ c15210_g10_i5.p1; TRISOR_ c11034_g1_i1.p1; TRISOR_ c15210_g10_i7.p1; TRISOR_ c11377_g1_i2.p1; TRISOR_ c10221_g1_i1.p1; TRISOR_ c13467_g1_i1.p1) and other homologous Kazal-type sequences from: *Panstrongylus chinai* (PANCHI_BBA30649.1); *Triatoma infestans* (TRIINF_ABR27937.1); *Triatoma brasiliensis* (TRIBRA_ABJ96351.1); *Hybomitra bimaculata* (HYBBIM_P84843.1); and *Tabanus yao* (TABYAO_ABX80080.1). The alignments indicate conserved residues in a blue scale background. The sequences are identified as described in the *Methods* section. The grey bar above indicates the Kazal domain region, with coloured asterisks showing the arrangement of the six conserved cysteines (C1:C5, C2:C4, C3:C6). Consensus sequence shows the frequency of amino acids in the multiple sequence alignment. The height of aminoacid symbols within the stack indicates the relative frequency of each amino acid at that position.

Here, 27 contigs, corresponding to 12.8% of the FPKM from the S category, were extracted as Kazal-type inhibitors (**Table 3**). Sialoproteome analysis identified 4 Kazal-type proteins, which corresponds to 1.4% of the total spectrum count (**Table 4**). Sequence identity of Kazal-type members from *T. sordida* and the triatomines *P. chinai, T. infestans, T. brasiliensis*, and the tabanids *H. bimaculata* and *Tabanus yao* were compared, showing all of them have one domain coding for six cysteine residues in a conserved arrangement of Kazal-type proteins C1:C5, C2:C4, C3:C6, with high variability in the remaining amino acids within the domain (**Figure 5**).

**Figure 6:**
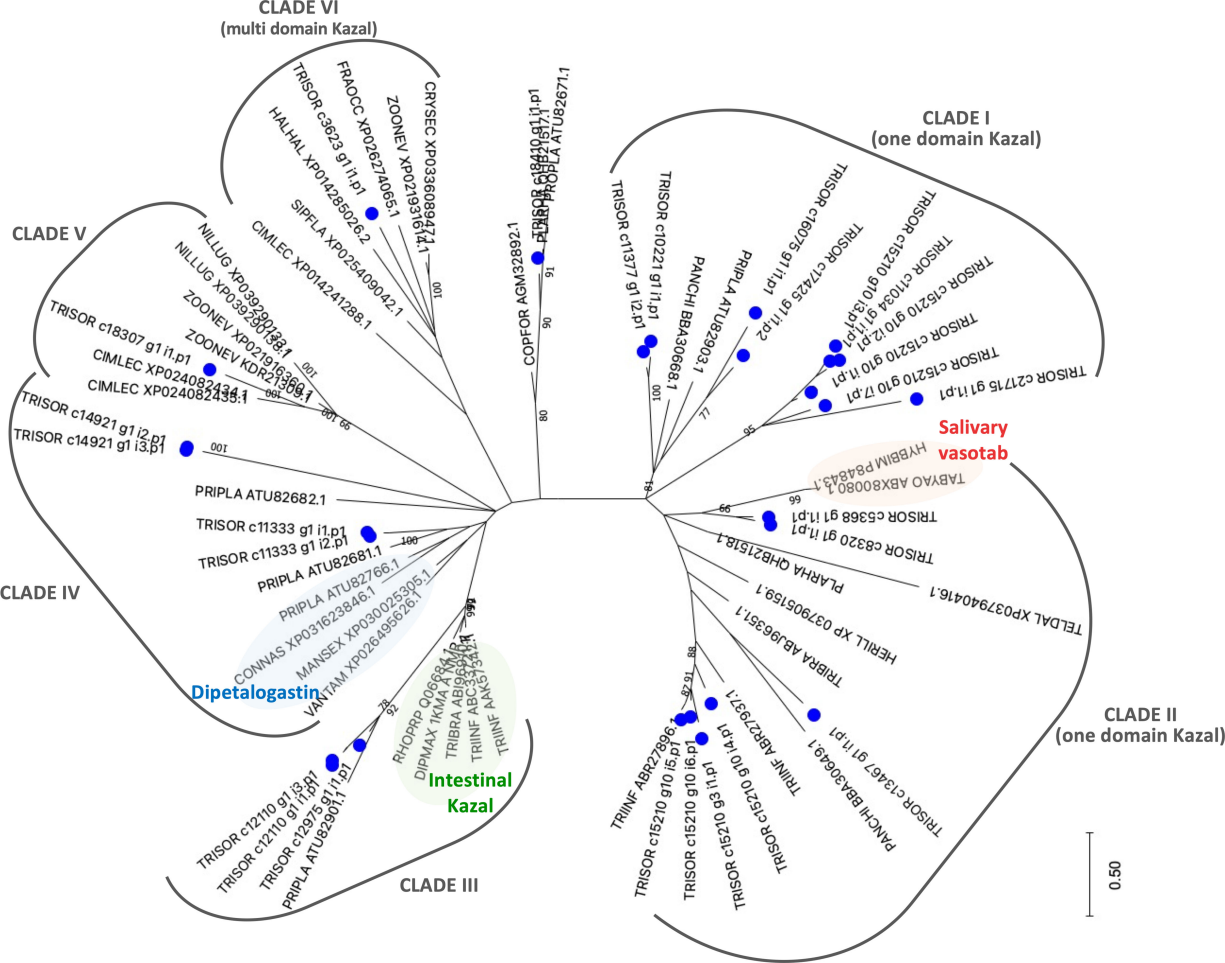
The phylogenetic tree of Kazal-type members from *Triatoma sordida* sialotranscriptome. The distance tree among trialysin sequences is derived from the alignment of *T. sordida* CDS extracted from the sialotranscriptome analysis and other Kazal-type sequences (extracted from the non-redundant protein database of the NCBI applying PSI-BLAST algorithm) identified as described in *Methods* section. The blue circle identifies sequences from *T. sordida*. The number at the nodes indicates the bootstrap values. The scale on bottom measure evolutionary distance in substitutions per amino acid.

Comparative phylogenetic analysis of *T. sordida* Kazal-type members and their homologous sequences found after iterative PSI-BLAST searches shows *T. sordida* Kazal-type proteins grouped within different clades. The results indicate *T. sordida* Kazal-type inhibitors are coded by different genes, and some are present in several isoforms. The variety of intra-domain, and also in N- and C-terminus residues suggest the folding structures of these proteins are versatile, allowing different binding sites to fit their respective target enzyme. Thus, they may perform different functions. While *T. sordida* Kazal-type protein from Clade VI may operate outside a feeding role, the members from Clades I, II and III (**Figure 6**), as suggested by their function in the intestines of triatomines and saliva from hematophagous arthropods, might work on inhibition of thrombin or vasodilation stimuli, impairing local host’s hemostasis.

#### 4.1.4 Enzymes

The analysis of *T. sordida* sialotranscriptome disclosed a variable set of enzymes comprising 3.8% of the FPKM from S category: Inositol phosphate phosphatase (INP-5) (FPKM = 2.0%); Salivary trypsin (FPKM = 1.1%); Metalloproteases (FPKM = 0.4%); 79 kDa salivary apyrase (FPKM = 0.3%); Nucleases (FPKM < 0.1%); Secreted Carboxylesterase (FPKM < 0.1%); Lysosomal aspartic protease (FPKM < 0.1%); and Lipases (FPKM < 0.1%) (**Table 3**). CDS encoding these enzymes are commonly found in Triatominae sialotranscriptomes. Here, the main enzymes with high transcript read counts are INP-5, salivary trypsin, metalloprotease and apyrase.

INP-5 members are abundant in transcriptome (**Table 3**), reaching 16.0% of spectrum count on proteome (**Table 4**). This feature is commonly observed in Triatominae sialotranscriptomes, but not in mosquitos or ticks (Santiago et al., 2020a). Based on its expression abundance, it is possible to infer the protein is secreted by SGs and play a relevant role in feeding performance. However, its function is puzzling, as inositol phosphates are important intracellular signalling molecules (Ribeiro et al., 2015). The activity of salivary trypsin (named triapsin) from *T. infestans* was already proposed as having a vasodilatory function mediated by PAR-2 peptide cleavage (Amino et al., 2001; Oliveira et al., 2021). In the same species, it was reported that salivary apyrase performs the hydrolysis of the ADP, an important agonist of the host’s platelet aggregation (Faudry et al., 2004; Faudry et al., 2006) and presents different isoforms (Charneau et al., 2007). The antiplatelet enzyme apyrase has been ubiquitously identified in saliva of triatomines (Ribeiro et al., 1998; Santiago et al., 2020a) and other hematophagous arthropods (Law et al., 1992; Ribeiro et al., 1998) measured the salivary apyrase activity among 24 triatomine species, including *T. sordida*, which showed larger ADPase than ATPase activity, Mn^2+^ and Co^2+^ dependence and 7.5 optimum pH. Apyrases show a variation in dependence on divalent cations and pH for optimum activity, suggesting their activity may derive from nonhomologous molecules (Ribeiro et al., 1998). Finally, the functional role of salivary metalloprotease in triatomines has not been reported to date, although it also may inhibit platelet aggregation and clot formation as has been reported for snake venom and tick (Francischetti et al., 2003; Kini and Koh, 2016).

#### 4.1.5 Ubiquitous Protein Families

Ubiquitous protein families’ groups comprise 3.1% of the FPKM from the S category and include the following subgroups: antigen-5 (Ag-5), conserved insect secreted protein and low-density lipoprotein receptor-related protein. Here, Ag-5 CDS is the fourth most expressed subgroup within the S category, accounting for 2.9% of the FPKM (**Table 3**). In addition, four Ag-5 proteins were identified in the sialoproteome, matching 5.9% of the total spectrum count (**Table 4**), suggesting the salivary mixture of *T. sordida* is made up of a remarkable diversity of anti-hemostatic mechanisms.

Ag-5 proteins are part of the CAP superfamily (cysteine-rich secretory proteins, antigen-5 and pathogenesis-related 1 proteins). It is a major allergenic component of Vespoidea venoms (Hoffman, 1993; King and Spangfort, 2000; Assumpção et al., 2008; Blank et al., 2020). The molecule is frequently found in saliva of blood feeders such as ticks and triatomines, although its contributions to the sting reaction still need to be uncovered (Ribeiro et al., 2004; Santos et al., 2007; ; Assumpção et al., 2008; Kato et al., 2010; Assumpção et al., 2011; Assumpção et al., 2012; Ribeiro et al., 2012b; Ribeiro et al., 2015; Santiago et al., 2016; Hernández-Vargas et al., 2017; Kato et al., 2017; Nevoa et al., 2018; Santiago et al., 2018; Blank et al., 2020; Mizushima et al., 2020; Santiago et al., 2020a). The function of Ag-5 salivary members from *T. infestans* and *D. maxima* has been reported (Assumpção et al., 2013). The molecule impairs platelet aggregation, ATP secretion, and thromboxane A_2_ generation by low doses of collagen, and *D. maxima* Ag-5 exhibited antioxidant activity, disrupting the host immune system (Gibbs et al., 2008; Assumpção et al., 2013; Blank et al., 2020).

### 4.2 Further Analysis of *Triatoma sordida* Sialome

**Figure 7:**
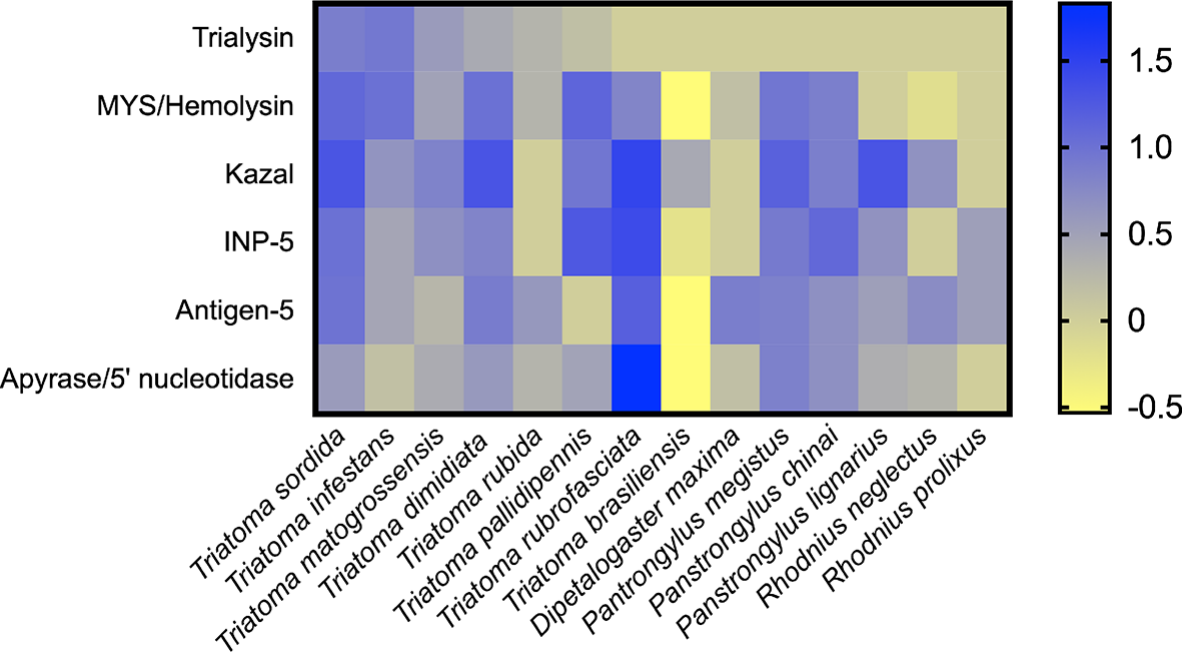
Heat map representing the expression profile of Triatominae salivary CDS. The heatmap shows the normalized CDS count of the main secreted families from Triatominae sialotranscriptomes. The differences among the CDS counts were statistically significant when compared by a Chi-square test (P < 0.001) (see **Supplementary Table 3**). Rows represent salivary families and columns represent the Triatominae species. The number of CDS frequencies were calculated for each species followed by log10 transformation. Blue-yellow scale: blue represents a high CDS count and yellow low represents low CDS count. The heatmap was created using GraphPad Prism software.

We compared the CDS count of the main expressed salivary families already reported in Triatominae sialotranscriptomes (Ribeiro et al., 2004; Assumpção et al., 2008; Assumpção et al., 2011; Assumpção et al., 2012; Ribeiro et al., 2012a; Ribeiro et al., 2015; Santiago et al., 2016; Hernández-Vargas et al., 2017; Kato et al., 2017; Nevoa et al., 2018; Santiago et al., 2018; Mizushima et al., 2020) (**Figure 7**). Note that the comparison was calculated using the CDS count previously reported using Sanger or Illumina sequencing technologies. In this context, only the most important families were considered, as those with low expression levels may not be represented in all sequencing results. In addition, this analysis represents the diversity of the extracted CDS, which does not reflect the expression abundance of each one. As a strategy for normalizing CDS counts, we used lipocalin as an internal standard, as it is enriched in all sialotranscriptomes investigated so far (Santiago et al., 2020a). The differences among the CDS counts of the species were statistically significant when compared by a Chi-square test (P < 0.001) (**Supplementary Table 3**). As expected, lipocalin is the most diverse family in all triatomine species analyzed (**Supplementary Table 3**). The resulting comparison shows that, frequently, Kazal-type is the second most diverse salivary family, a feature observed in *T. sordida*, *T. matogrossensis*, *T. dimidiata*, *T. brasiliensis*, *P. megistus* and *Panstrongylus lignarius*. Higher expression of CDS coding for MYS/hemolysin is observed in *T. sordida*, *T. infestans*, *T. matogrossensis*, *T. dimidiata*, *T. pallidipennis*, *P. megistus* and *P. chinai*. Kazal-type family is enriched in *T. sordida*, *T. dimidiata*, *T. rubrofasciata* and *P. megistus*, whereas trialysin is enriched in *T. sordida*, *T. infestans* and *T. matogrossensis*. The overall results suggest that each Triatominae species has a distinct salivary expression signature.

An insight into the potential protein complexes found in triatomines saliva was recently unveiled, and untraditional proteins were highlighted (Santiago et al., 2020b). It has been suggested the sialocomplexomes (salivary protein complexes) are arranged to form multifunctional modules that enhance the counteraction of host’s response and blood acquisition. Here, *T. sordida* sialoproteome reveals some proteins found in the reported sialocomplexes (Santiago et al., 2020b). The heat shock cognate protein 70 kDa (HSP70), vitellogenin lipoprotein and apolipophorin are among them. The role of HSP70 is not clear, but in ticks, it was proposed the molecule interferes with host fibrinogenolysis at the bite site (Vora et al., 2017). Apolipophorin and vitellogenin are hemolymph lipid transporter proteins, but it was proposed both may perform alternative functions. The former may mediate immune responses, while the second transport dietary lipids (Kim et al., 2004; Smolenaars et al., 2007; Zdybicka-Barabas and Cytryńska, 2013).

## 5 Conclusions

Sialome descriptions have already been performed in Triatominae species of the genera *Dipetalogaster, Rhodnius, Triatoma* and *Panstrongylus*, revealing a shared set of protein families that have specific targets in the hemostatic system of the vertebrate host and work in a pleiotropic manner (Santiago et al., 2020a). Here, the *T. sordida* sialome reveals the lipocalin gene family to be abundant and evolving through lineage-specific expansion (gene duplication), a remarkable feature of Triatominae species. An interesting feature in the *T. sordida* sialome is the high abundance of trialysin and Kazal-type members. The search for abundant and/or underexploited food resources may explain the variation observed in the salivary protein mix, driving host preference selective pressure in this peridomestic *T. sordida* population. It has been suggested that the blood-feeding behaviour in reduviid bugs is polyphyletic and evolved several times from different predacious or hemolymph-sucking ancestors (Schofield and Galvão, 2009). Triabin, trialysin, MYS/hemolysin, Kazal-type members, and CAP/Ag-5 members, among others, were recruited into the venom of Hemiptera before the switch of insectivory to hematophagy, acquiring new functions through accelerated evolution of feeding specialization (Walker et al., 2017).

Here we reported the protein family members from *T. sordida* sialome, making a comprehensive mapping of novel anti-hemostatic compounds that can be exploited for therapeutic use. Our work contributes to the advances in the knowledge of hematophagous salivary molecules, giving insight into the *T. sordida* sialome and the understanding the evolution of salivary/venom protein families in Hemiptera. Some questions arose from our results: Which variables may play a role in the modulation of triatomine salivary protein abundance? How do they influence blood-feeding and/or *T. cruzi* infection processes?

## Data Availability Statement

The datasets presented in this study can be found in online repositories. The names of the repository/repositories and accession number(s) can be found in the article/**Supplementary Material**.

## Author Contributions

CA, PS, and JS designed the study. IS was responsible for triatomine collection. YP, KB, SS, SC, SM, WS, MS, and CS were responsible for the assays and the acquisition of data. JR, YP, PS, and CA analyzed the experimental data. YP, PS, and CA wrote the manuscript. PS, CA, SC, IB, JR, and JS were the major contributors in revising the manuscript. All authors read, edited, and approved the final manuscript.

## Funding

This work was supported by grants and fellowships awarded by the Coordenação de Aperfeiçoamento de Pessoal de Nível Superior (CAPES, grant 923/18 CAPES-COFECUB), Conselho Nacional de Desenvolvimento Científico e Tecnológico (CNPq, INCT-MCTI/CNPq/CAPES/FAPs 16/2014), Fundação de Amparo à Pesquisa do Distrito Federal (FAP-DF, grants 0193.001802/2017 and 00193-00000229/2021-21), Financiadora de Estudos e Projetos (Finep, grant CT-Infra 0439/11). JR was supported by the Intramural Research Program of the National Institute of Allergy and Infectious Diseases (Vector-Borne Diseases: Biology of Vector Host Relationship, Z01 AI000810-19). Because JR is a government employee and this is a government work, the work is in the public domain in the United States. Notwithstanding any other agreements, the NIH reserves the right to provide the work to PubMedCentral for display and use by the public, and PubMedCentral may tag or modify the work consistent with its customary practices. Rights can be established outside of the United States subject to a government use license. YP was recipient of Ph.D. grant supported by the Coordenação de Aperfeiçoamento de Pessoal de Nível Superior (CAPES).

## Conflict of Interestt

The authors declare that the research was conducted in the absence of any commercial or financial relationships that could be construed as a potential conflict of interest.

## Publisher’s Note

All claims expressed in this article are solely those of the authors and do not necessarily represent those of their affiliated organizations, or those of the publisher, the editors and the reviewers. Any product that may be evaluated in this article, or claim that may be made by its manufacturer, is not guaranteed or endorsed by the publisher.
