# Supplementary material for: An Integrative Sialomic Analysis Reveals Molecules From Triatoma sordida (Hemiptera: Reduviidae)
Source: Front Cell Infect Microbiol. 2022 Jan 3;11:798924. doi: 10.3389/fcimb.2021.798924 (PMC8762107; doi:10.3389/fcimb.2021.798924)
Supplement: Supplementary file 1 [file DataSheet_1.pdf]

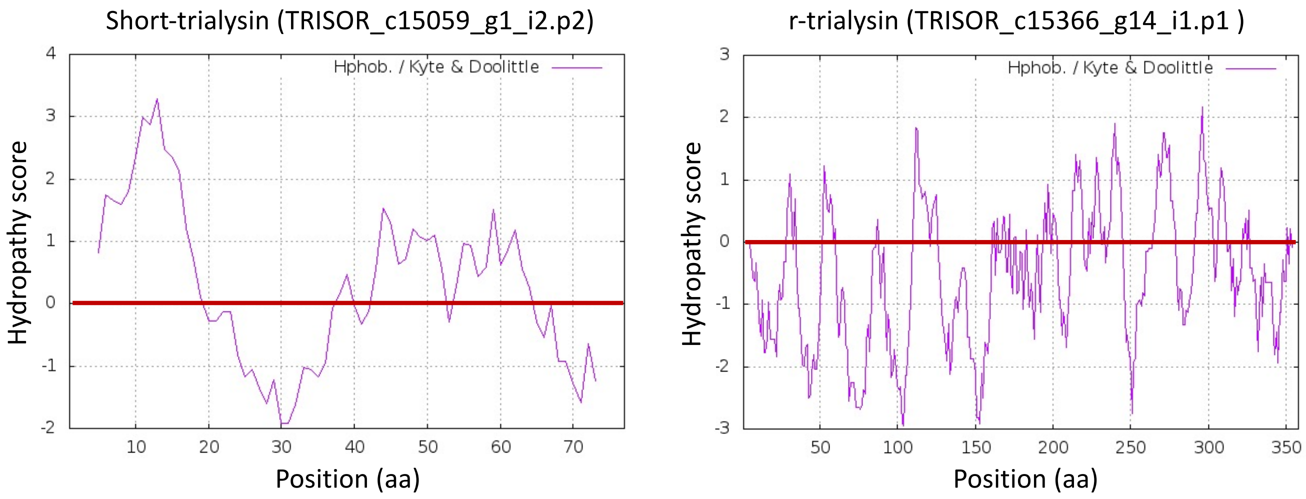

Supplementary Figure 1. Kyte and Doolittle hydrophobicity plot of short-trialysin and r-trialysin from *Triatoma sordida* sialotranscriptome. Plot was created using the ExPASy Protscale tool (http://web.expasy.org/protscale/). Hydrophobic amino acids are displayed above the zero line, and hydrophilic amino acids are below.
